# Developing a curriculum to improve cardiology fellows’ training in pregnancy and cardiovascular disease

**DOI:** 10.1186/s12909-022-03228-7

**Published:** 2022-03-10

**Authors:** Benjamin Maxner, Barinder Hansra, Diana Sibai, Sheikh Moinul, Leslie Panella, Jeannine Jeha, Catherine Fiore, Tina Dumont, Julianne Lauring, Gerard Aurigemma, Colleen M. Harrington, Lara C. Kovell

**Affiliations:** 1 University of Massachusetts Medical School, Worcester, MA USA; 2 Department of Critical Care Medicine, University of Pittsburgh Medical Center, PA Pittsburgh, USA; 3 Division of Cardiovascular Medicine, Department of Medicine, University of Massachusetts Medical School, Worcester, MA USA; 4 Department of Medicine, University of Massachusetts Medical School, Worcester, MA USA; 5 Division of Maternal-Fetal Medicine, Department of Obstetrics and Gynecology, University of Massachusetts Medical School, Worcester, MA USA

**Keywords:** Cardiology, Fellowship, Pregnancy, Cardiovascular disease

## Abstract

**Background:**

Exposure to pregnant women with cardiovascular disease (CVD) during cardiology fellowship training is limited and without a standard curriculum in the United States. The authors sought to evaluate a dedicated curriculum to teach management of CVD in pregnancy to improve general cardiology fellowship training.

**Methods:**

The authors developed a dedicated CVD in pregnancy curriculum for the general cardiology fellows at a large academic medical center in the fall of 2019. Fellows’ knowledge was assessed via a board-style examination and exposure and attitudes related to the care of pregnant women with CVD were evaluated with a needs assessment questionnaire before and after the curriculum.

**Results:**

Of the 17 fellows who participated in the curriculum, 12 completed the needs assessment pre-curriculum and 9 post-curriculum. The mean (SD) number of pregnant women with CVD cared for by each fellow in the inpatient and outpatient settings were 0.75 (1.29) and 0.56 (0.73), respectively. After the curriculum, all fellows reported awareness of available resources to treat pregnant women with CVD, while a majority disagreed that they receive regular exposure to pregnant patients with CVD in their training. The authors observed significant increases in fellows’ confidence in their knowledge of normal cardiovascular physiology of pregnancy, physical exam skills, and ability to care for pregnant women with valvular disease and arrhythmias from pre to post-curriculum. A total of 15 fellows completed the board-style exam pre-curriculum and 15 post-curriculum. Fellows’ performance on the board-style examination improved slightly from before to after the curriculum (64.0 to 75.3% correct, *p* = 0.02).

**Conclusions:**

A dedicated curriculum improved cardiology fellows’ knowledge to recognize and treat CVD in pregnancy and improved confidence in caring for this unique patient population.

## Background

Women in the United States (US) have the highest rate of maternal mortality compared to other high-income countries and this rate is rising, with cardiovascular disease (CVD) as the leading cause of death [1]. Perinatal providers are tasked with having a comprehensive understanding of normal and abnormal cardiac physiology in order to understand the hemodynamic changes that occur in pregnancy. Cardiologists are central to the care of women with CVD who are planning to become pregnant or are already pregnant. This includes women at high risk for complications during pregnancy, such as those with underlying cardiomyopathies, aortopathies, valvular disease, arrhythmias, or congenital heart disease [2]. CVD in pregnancy is associated with increased pregnancy complications and mortality at later stages in life [2]. For these reasons, it is important for women with risk factors of CVD and established CVD to be followed closely by cardiology throughout pregnancy and beyond.

Despite these unfortunate trends in women’s health, there is no standard curriculum or minimum requirements for cardiology fellows in the US to learn about the management of CVD in pregnancy. The Cardiology-specific Accreditation Council for Graduate Medical Education (ACGME) requires all cardiology fellows to be familiar with the management of pregnancy and heart disease [3] but does not have specific curricular recommendations. Furthermore, these skills are not taught or assessed in a standard fashion but rather dependent on individual experience and faculty instruction which can lead to heterogeneity in fellow skills and experience. It is also unknown what current exposure US cardiology fellows have to women with CVD who are planning to become or are already pregnant.

In response to these gaps, we sought to develop and evaluate a multi-disciplinary pregnancy and CVD curriculum for cardiology fellows and to assess their knowledge, attitudes, and exposure to this unique population in order to improve cardiology fellowship training.

## Methods

We created an interactive, multi-disciplinary pregnancy and CVD lecture series that spanned the course of 4 weeks in the fall of 2019 for the general cardiology fellows at the University of Massachusetts Medical Center. IRB approval was not required for this study since no personal information was disclosed and results of the study’s surveys did not affect the fellows’ standing in the program. This lecture series was led by expert physicians in their respective fields including general cardiology, electrophysiology, maternal fetal medicine, and hematology. The lecture topics covered included physiological and hemodynamic changes during pregnancy, arrhythmias, valvular disease, pre-conception counseling, pre-delivery planning, cardiomyopathies, anti-coagulation, hypertension, and a dedicated lecture on the safety of cardiovascular medications in pregnancy (Table 1)**.** Fellows were encouraged to actively participate in lectures by asking questions and working through relevant case-based learning presentations.

**Table 1:** Lecture topics and corresponding objectives presented in fall curriculum

Lecture Topic	Objectives
Introduction to Pregnancy and Physiology	• Review basics of cardiovascular physiology. • Understand normal physiological changes during pregnancy. • Review case-based scenarios of everyday practice.
Pregnancy and Heart Disease: Hypertensive Disorders of Pregnancy	• Review the definitions of hypertensive disorders of pregnancy. • Describe the rationale for treatment of hypertension in pregnancy. • Understand the long-term implications of preeclampsia on cardiovascular healthy.
Pre-Conception Counseling	• Review the hemodynamic changes during pregnancy, normal and abnormal physical exams and symptoms. • Describe the rationale for pre-conception counseling and tools we have to estimate risk. • Understand the cardiac contraindications for pregnancy.
Pre-Delivery Planning	• Outline an approach to preconception planning for women with cardiovascular disease. • Describe the appropriate types of monitoring required during pregnancy. • List the considerations that need to be covered when making recommendations for delivery planning.
Medication Use and Safety in Pregnancy	• Name the factors that affect teratogenic potential of a medication during pregnancy and lactation. • Discuss the pregnancy and lactation-related risks of the major classes of medications used for cardiovascular disease. • List potential resources for up to date information on medication use in pregnancy and lactation.
Valvular Disease in Pregnancy	• Describe the normal hemodynamic physiologic changes associated with pregnancy. • Review the clinical findings and echocardiographic findings seen in mitral and aortic stenosis. • Discuss the effect of pregnancy on mitral and aortic stenosis and their management. • Management of anticoagulation for mechanical valves in pregnancy. • Discuss preconception counseling regarding valve type and the risk during pregnancy.
Arrythmias in Pregnancy	• Most common arrhythmia in pregnancy – modern day • Cardiac arrest in pregnancy a. Timing of cesarean delivery in cardiac arrest b. Mode of effective CPR in pregnancy • Anticoagulation in pregnancy a. Mechanical Valves b. Atrial fibrillation • Safe antiarrhythmic drugs (AADs) a. Beta Blockers b. Calcium Channel Blockers c. Other AADs • Contraindicated cardiac medications.

We assessed knowledge via board-style examination questions adapted from the American College of Cardiology In-Training Exam and evaluated exposure and attitudes through a needs assessment questionnaire before and after the curriculum. The needs assessment questionnaire focused on fellows’ confidence in treating pregnant patients with CVD, their clinical exposure to this unique population during their training, and their understanding of the resources available to them at our institution. Each needs assessment questionnaire prompt used a 5-point Likert scale to rate agreement or disagreement with a statement about the management of CVD during pregnancy. We grouped survey responses on the 5-point Likert scale into three larger categories (strongly disagree or somewhat disagree, neutral, somewhat agree or strongly agree) and the proportion of fellows selecting each response were compared between timepoints using Fisher’s exact test. We compared board-style examination results by content category and overall score using two-sample t-tests. A *p*-value of < 0.05 was considered statistically significant for all comparisons. We completed all analyses using R version 3.5.1.

At the end of the curriculum, we conducted a town-hall style discussion with the general cardiology fellows to discuss the positive aspects and areas where improvement was needed. In addition, we discussed future lecture topics of interest along with potential changes to improve future iterations.

## Results

**Fig. 1:**
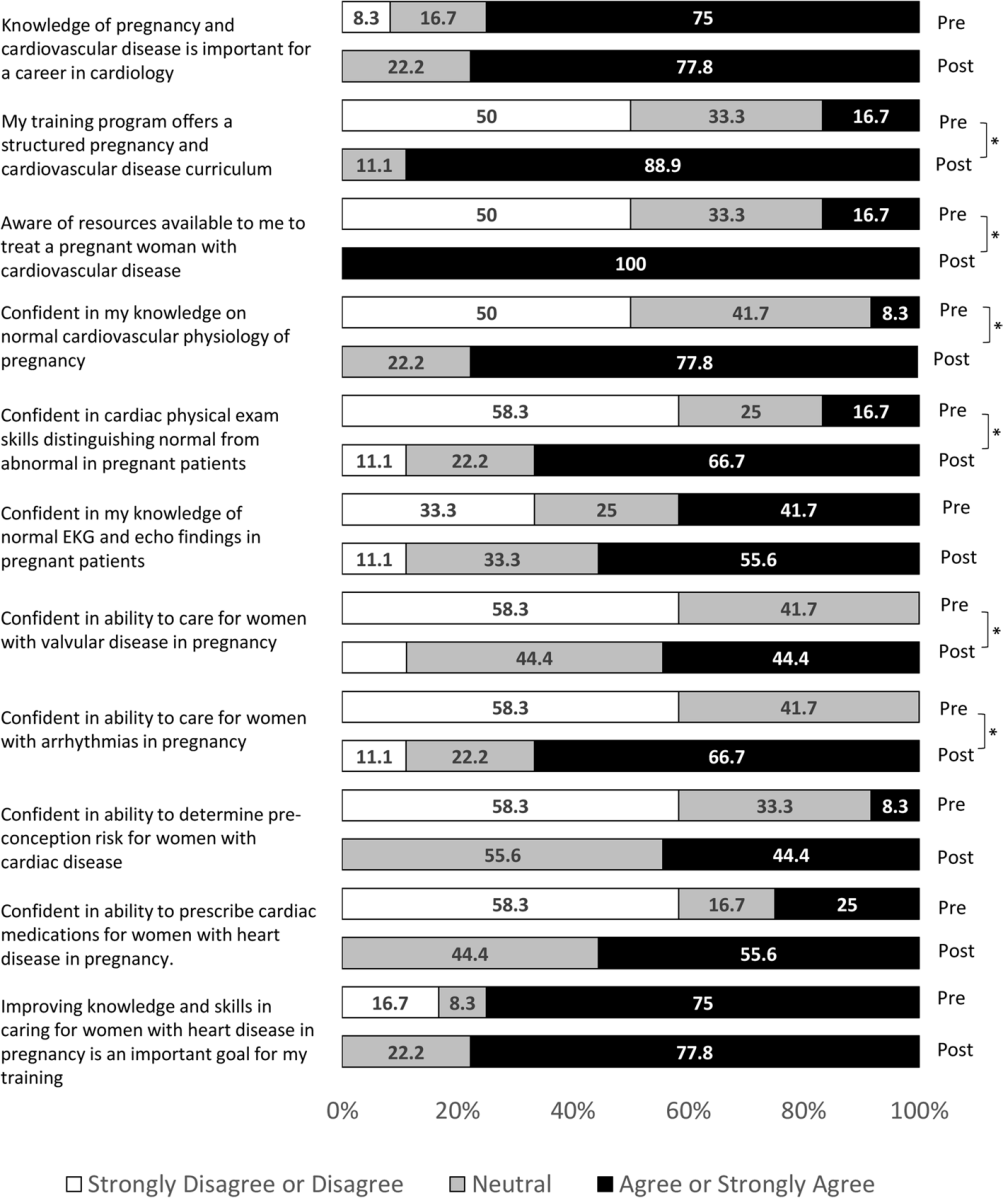
Pre- and post-curriculum needs assessment responses among cardiology fellows (*n* = 12 for pre-curriculum; *n* = 9 for post-curriculum). *Indicates significant pairwise comparison for level of agreement between pre- and post-curriculum (*p* < 0.05)

Of the 17 general cardiology fellows who participated in the curriculum, 12 completed the needs assessment pre-curriculum and 9 completed the assessment post-curriculum. The mean (SD) number of pregnant women with cardiovascular disease cared for by fellows in the inpatient and outpatient setting were 0.75 (1.29) and 0.56 (0.73), respectively. After the curriculum, 88.9% of the fellows agreed or strongly agreed that their program offered a structured pregnancy and heart disease curriculum, while all the fellows agreed or strongly agreed that they were aware of resources available to them to treat pregnant women with cardiovascular disease (Fig. 1). However, 78% disagreed or strongly disagreed with the statement “I am exposed to pregnant patients with suspected or known cardiovascular disease regularly in my training”. In addition, there were significant increases in the proportion of fellows reporting agreement or strong agreement with their confidence in their knowledge of normal cardiovascular physiology of pregnancy, their physical exam skills distinguishing normal from abnormal in pregnant patients, and their abilities to care for pregnant women with valvular disease and arrhythmias (All *p* < 0.05) (Fig. 1).

**Fig. 2:**
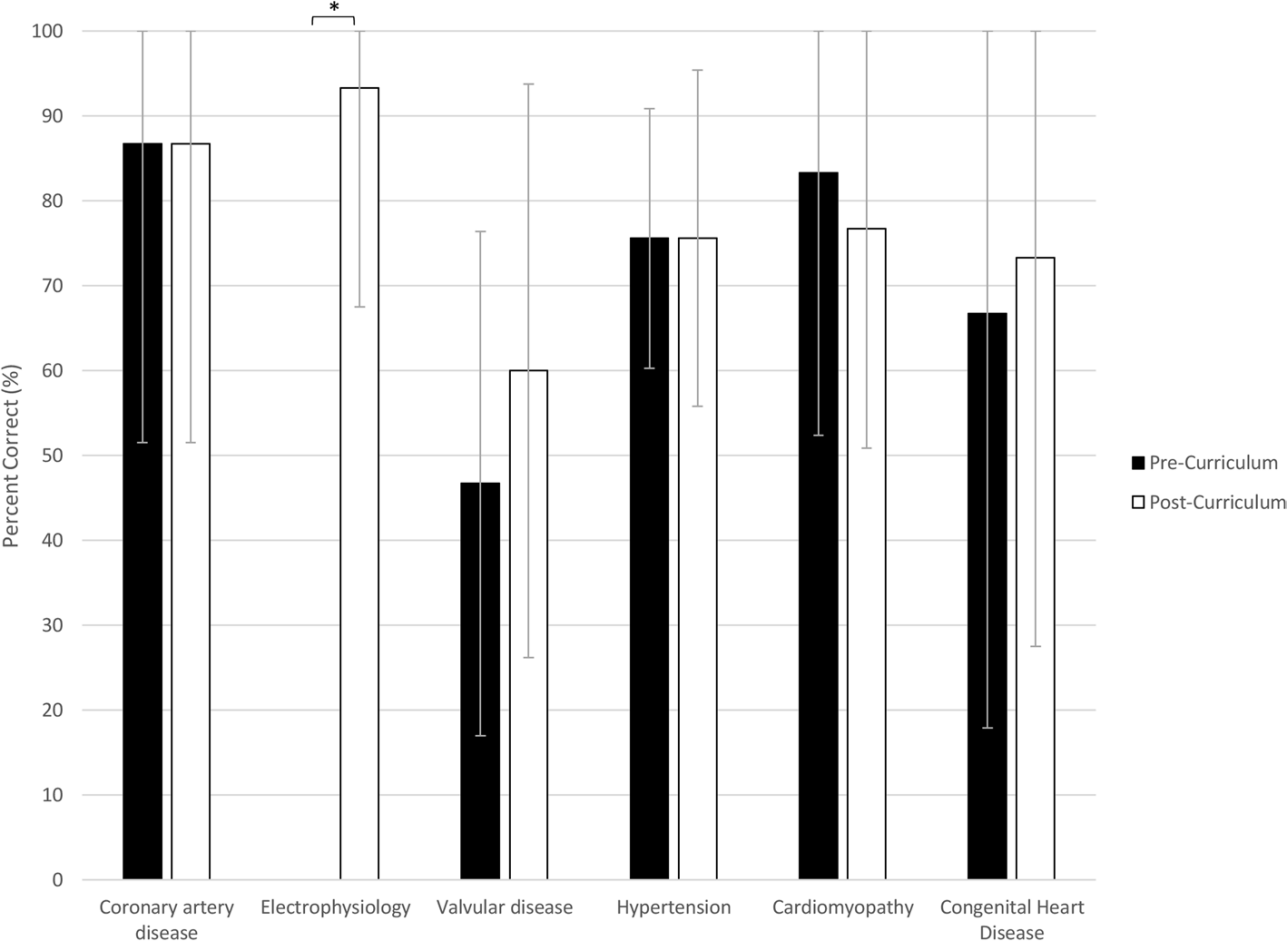
Pre-curriculum and post-curriculum board-style examination scores among cardiology fellows by content area. * Indicates significant pairwise comparison between pre- and post-curriculum *p* < 0.01

In regard to the board-style examination, a total of 15 fellows completed the exam pre-curriculum and 15 post-curriculum. Fellows’ performance on the examination significantly improved from 64.0% correct pre-curriculum to 75.3% correct post-curriculum (*p* = 0.02). Specifically, fellows scored significantly higher on electrophysiology after the curriculum with an increased focus on case examples and medications (0% vs 93.3% correct, *p* < 0.01). There were no significant changes in performance on the cardiomyopathy, coronary artery disease, valvular disease, hypertension, and congenital heart disease questions (Fig. 2).

## Discussion

In this curriculum-based prospective study, we found that regular cardiology fellow exposure to pregnant women with CVD is low at our medical center. Our dedicated curriculum improved cardiology fellows’ knowledge and attitudes towards caring for this vulnerable population. We observed increased confidence among fellows in their physical exam skills distinguishing normal from abnormal in pregnant patients and in their abilities to care for pregnant women with valvular disease and arrhythmias.

In the current cardiology training model, multiple gaps exist within fellows’ clinical exposure to pregnant women with CVD and the disbursement of relevant clinical knowledge and skills. To address these gaps, a curriculum and training program is needed where cardiology fellows develop comprehensive knowledge and skills to recognize the normal and abnormal changes occurring in pregnancy. However, the ACGME does not have a standardized education series for trainees on this topic. The ACGME does require cardiology fellows to be familiar with management of pregnancy and heart disease, specifically stating “fellows must be able to provide patient care that is compassionate, appropriate, and effective for the treatment of health problems and the promotion of health in heart disease in pregnancy.” [3] Unfortunately, specific topics or disease processes are not highlighted. Over the past 20 years, the American College of Cardiology (ACC) has recognized and supported the utilization of the Core Cardiology Statement (COCATS) to help define the academic, clinical, and technical skills required for proficiency in adult cardiology [4]. Despite the presence of core competencies, the COCATS do not define structured curriculum for cardiology fellows across the country to study pregnancy-related CVD. In fact, COCATS is a product that results entirely from expert consensus, rather than educational research [5]. In contrast, the American Board of Obstetrics and Gynecology along with the Maternal-Fetal Medicine ACGME have detailed educational objectives for their trainees including but not limited to: describing normal cardiac function in pregnancy, auscultatory and hemodynamic changes, and interpretation and expected results from maternal echocardiography in pregnancy [6]. There is also a strong emphasis on identifying preexisting maternal cardiac conditions, which may adversely affect cardiac function during and after pregnancy, such as mitral stenosis, aortic regurgitation, and right-to-left shunts [6, 7]. This study aimed to address these gaps by offering a standardized curriculum taught by experts in multiple specialties to increase fellows’ exposure to cases of pregnant women with CVD and improve the acquisition of clinical knowledge and skills necessary to provide better care.

Prior studies have shown physicians are spending less time at the bedside, which has contributed to a decline in physical diagnosis and cardiopulmonary examination skills [8]. A report from nine maternal mortality review committees also identified systems of care and provider factors contributing to maternal mortality, with lack of knowledge and inadequate training as major dominant themes. Specifically, provider factors represented 26%, 37%, and 62% of the critical factors responsible for maternal cardiovascular deaths, maternal cardiomyopathy deaths, and preeclampsia and eclampsia deaths, respectively [9]. In our study, fellows’ confidence in their physical exam skills and ability to care for pregnant women with CVD was low at baseline. It was encouraging that fellows who participated in the curriculum reported significant increases in their confidence to distinguish abnormal from normal findings on physical exam in pregnant women and to care for pregnant women with arrhythmias or valvular disease. With maternal mortality on the rise and CVD being the leading cause of maternal death [1], there is an urgent responsibility of cardiology fellowship programs to develop systematic methods of training fellows who are equipped with the knowledge, skills, and resources needed to treat these patients. Increasing cardiology fellows’ exposure to these patients and expanding their knowledge base and clinical skillset can significantly impact the health of pregnant women with CVD throughout the U.S. [5]

Recently, both the European Society of Cardiology and the ACC have endorsed a multidisciplinary team approach for women with CVD with moderate or high-risk of complications during pregnancy. This team should aid in counseling and management prior to conception and during pregnancy, delivery, and the postpartum period. The minimum team requirements are a cardiologist, obstetrician, and anesthesiologist, all with expertise in the management of high-risk pregnancies in women with heart disease [2, 10]. In theory, any cardiology fellows going into practice could be a part of this crucial team. However, without a well-trained, proficient cardiologist familiar with CVD and complications during pregnancy, less than ideal care may be delivered, which can contribute to poor outcomes. Mirroring the multidisciplinary nature of the care team, educational initiatives should include of all specialties involved in the treatment of this vulnerable population in order to leverage the unique expertise of each discipline [11]. Our curriculum included lectures taught by multiple members of this interdisciplinary team and focused on care from pre-pregnancy counseling through postpartum follow-up. Reflecting this broad exposure, all fellows reported increased awareness of the resources available to them to treat pregnant women with CVD, which should allow them to coordinate care more efficiently among team members in the future.

The lectures delivered in the curriculum were given over the span of 4 weeks and the cardiology fellows showed improvement in their knowledge base after the completion of the lecture series. There is limited data regarding effective educational strategies for trainees, and even less data looking at changing the duration of lectures to improve knowledge retention [12]. The primary goal of education is to promote long-term knowledge storage and retrieval [13]. A small study suggests dispersed delivery (DD) of lectures versus mass delivery (MD) is more effective at long term knowledge retention and retrieval [13]. This led us to deliver our curriculum over a short, dedicated time period but not all in 1 day to promote repetition and ensure long-term knowledge retention needed for critical thinking and application of knowledge to clinical scenarios. Our lecturers also focused on encouraging audience participation, providing case-based learning, and incorporating questions – all effective strategies to engage trainees in critical thinking and analysis of complex medical conditions [14]. With the strategic design of this curriculum, fellows’ retention of the information and performance on the board-style examination significantly improved from before to after the lecture series.

A critical component of implementing training programs is accurately assessing their impact. A widely utilized process to assess educational programs is the Kirkpatrick Model, which utilizes a 4-level model of increasing complexity from 1) reaction, 2) learning, 3) behavior, and 4) result [15]. Our curriculum reached level 2 in the Kirkpatrick model based on the results of the board-style examination and the needs assessment, with fellows’ increased knowledge and confidence in treating pregnant patients with CVD. However, we did not evaluate how the curriculum may have impacted their clinical practice and performance. Improving our curriculum to be operating at Kirkpatrick stage 4 would have a larger impact at an institutional and societal level [14]. With increased exposure to these patients in a clinical environment and the potential for simulation-based learning scenarios, future curriculum development can incorporate objective measures of how trainees’ improved knowledge and confidence translate to clinical skills and outcomes.

Our study has several limitations. This was a single center experience, so the results may not be applicable to other institutions. However, our curriculum can serve as a starting point for other cardiology fellowship programs. We were not able to capture 100% participation in our assessments of the lecture series and curriculum, which could have made the findings more robust. Additionally, we cannot rule out that the low score on the electrophysiology assessment is not partly due to a flaw in the question itself, and self-teaching could have contributed to the improvement on the post-curriculum assessment. As a result, the significance in the fellows’ improved performance in this topic relative to the others may be overestimated. It is also important to highlight that unlike electrophysiology, some topics in the board style examination did not have corresponding lectures dedicated to these topics. This could explain the marked improvement in electrophysiology and support curriculum changes that include more comprehensive instruction on topics with less notable improvement on the examination. Furthermore, we have not conducted testing to assess long-term knowledge retention amongst the cardiology fellows.

Moreover, fellows were not assigned the same identifier when taking the needs assessment and board style examination pre and post curriculum. As a result, inferences on improvements that occurred on an individual basis after the curriculum was introduced cannot be made. While we were able to have physician faculty from several specialties involved in lecture presentations, this curriculum lacked the engagement of team members from other health professions such as pharmacists, social workers, or nurse-midwives that could have contributed different perspectives and added nuance important to recognize when providing care to pregnant women with CVD. Finally, a major limitation in this study is the lack of clinical skills assessments, which prevents us from evaluating the impact of fellows’ improved knowledge and confidence on clinical outcomes in caring for this population.

Feedback from the needs assessment and post-curriculum town-hall discussion have focused our next steps – to address the low exposure to pregnant women with CVD during fellow training. On outpatient rotations, fellows will participate in our recently established Pregnancy and Heart Disease Clinic to increase exposure. We have regularly presented cases of pregnant women with CVD to the fellowship’s weekly case discussion and plan to work with the program leaders to incorporate simulation model learning. With the recent COVID-19 global pandemic, many fellowship programs have suspended in-person conferences and transitioned to virtual learning. Increased familiarity with platforms such as Zoom (Zoom Video Communications, San Jose, California), and others allow sharing of patient-level data through a Health Insurance Portability and Accountability Act–compliant server making it easier to incorporate remote learning and multidisciplinary team discussion [16]. We plan to expand our curriculum’s impact by providing live or on-demand formats that trainees in cardiology along with internal medicine, obstetrics and gynecology, maternal-fetal-medicine, and emergency medicine can access. Doing so will ensure the continuation of a multidisciplinary approach in engaging trainees with educational material and exposure to pregnancy and CVD to prevent loss of knowledge.

## Conclusions

Managing pregnant women with CVD is a complex process that requires multidisciplinary care, including a cardiologist, obstetrician, and anesthesiologist. Regular cardiology fellow exposure to pregnant women with CVD is low at our large, academic medical center, and our focused curriculum on pregnancy and CVD improved fellows’ knowledge and confidence. Although maternal mortality rates are rising, current pregnancy and CVD training and exposure for cardiology fellows remain inadequate. Standardized curricula must be developed in order to fill these critical knowledge gaps and improve outcomes for this vulnerable population.

## Data Availability

The datasets used and analyzed during the current study are available from the corresponding author on reasonable request.

## Abbreviations

CVD: Cardiovascular disease
US: United States
ACGME: Accreditation Council for Graduate Medical Education
ACC: American College of Cardiology
COCATS: Core Cardiology Statement
DD: Dispersed Delivery
MD: Mass Delivery
